# Artificial Intelligence-Based Distinction of Actinic Keratosis and Seborrheic Keratosis

**DOI:** 10.7759/cureus.58692

**Published:** 2024-04-21

**Authors:** Shreya Reddy, Dinesh Giri, Rakesh Patel

**Affiliations:** 1 Biomedical Sciences, Creighton University, Omaha, USA; 2 Research, California Northstate University College of Medicine, Elk Grove, USA; 3 Internal Medicine, East Tennessee State University Quillen College of Medicine, Johnson City, USA

**Keywords:** dermatological imaging, lesion classification, artificial intelligence (ai), seborrheic keratosis, actinic keratosis

## Abstract

Actinic keratosis (AK) and seborrheic keratosis (SK) represent prevalent dermatological conditions with distinct clinical characteristics and potential health implications. This article investigates recent strides in dermatological diagnostics, centered on the development and application of artificial intelligence (AI) technology for discerning between AK and SK. The objective of this study is to develop and evaluate an artificial intelligence (AI) model capable of accurately distinguishing between stage one and stage two gastric carcinoma based on pathology slides. Employing a dataset of high-resolution images obtained from Kaggle.com, consisting of 1000 AK and 1000 SK images, a novel AI model was trained using cutting-edge deep learning methodologies. The dataset underwent meticulous partitioning into training, validation, and testing subsets to ensure robustness and generalizability. The AI model showcased exceptional proficiency in distinguishing AK from SK images, attaining notable levels of accuracy, precision, recall, specificity, F1-score, and area under the curve (AUC). Insights into the etiology and clinical ramifications of AK and SK were presented, emphasizing the critical significance of precise diagnosis and tailored therapeutic approaches. The integration of AI technology into dermatological practice holds considerable potential for enhancing diagnostic precision, refining treatment decisions, and elevating patient outcomes. This article underscores the transformative impact of AI in dermatology and the importance of collaborative efforts between clinicians, researchers, and technologists in advancing the realm of dermatological diagnosis and care.

## Introduction

Actinic keratosis (AK) and seborrheic keratosis (SK) are frequently encountered dermatological conditions that exhibit clear differences in their clinical appearances. AK, also known as solar keratosis, is a precancerous skin lesion caused by prolonged sun exposure, typically appearing as rough, scaly patches on sun-exposed areas such as the face, scalp, and hands [1]. SK, on the other hand, is a benign skin growth characterized by waxy, elevated lesions that often resemble warts and tend to develop on areas of the body with high sebaceous gland activity, such as the face, chest, and back [2]. Globally, it is estimated that millions of individuals are affected by these conditions, with AK being particularly prevalent in fair-skinned individuals over the age of 40 and SK being more common among older adults [3,4].

Actinic keratosis (AK) evolves as a consequence of prolonged and cumulative exposure to ultraviolet (UV) radiation, spanning many years, which triggers aberrant growth in the keratinocytes residing within the epidermal layer of the skin [5]. This prolonged UV exposure induces damage to the DNA of skin cells, instigating mutations that disrupt normal cellular processes and facilitate the emergence of AK lesions [6]. The intricate interplay between environmental factors, such as UV radiation, and genetic predisposition contributes to the multifaceted pathogenesis of AK [7]. Individuals with fair skin, advanced age, and a history of recurrent sun exposure or sunburns are particularly susceptible to developing AK due to their heightened vulnerability to UV-induced DNA damage [5]. Furthermore, the risk of AK progression to invasive squamous cell carcinoma underscores the critical importance of early detection and intervention strategies [8]. If left untreated, AK lesions may evolve into malignant tumors, emphasizing the imperative for proactive screening and treatment modalities to mitigate the potential health risks associated with this precancerous condition [8].

Conversely, seborrheic keratosis arises from the uncontrolled proliferation of keratinocytes within the epidermal layer, leading to the formation of benign growths with a characteristic appearance akin to warts on the skin's surface [9]. Although the precise etiology of SK remains elusive, advancing age, genetic predisposition, and exposure to environmental factors such as sunlight are postulated to contribute to its development [9]. The gradual accumulation of genetic alterations and cellular changes over time underscores the complex pathogenesis of SK [9]. Unlike AK, SK lesions are predominantly benign and do not harbor the inherent risk of malignant transformation [9]. However, despite their benign nature, SK lesions can pose cosmetic concerns for affected individuals, potentially impacting their quality of life [10]. SK lesions can resemble more serious dermatological conditions, including squamous cell carcinoma, basal cell carcinoma, and other forms of skin cancer [9]. These similarities underscore the significance of accurate diagnosis and differentiation to prevent unnecessary anxiety and ensure appropriate management strategies [9,10]. This highlights the importance of comprehensive clinical evaluation and histopathological examination to distinguish SK from other dermatological conditions and guide optimal treatment approaches.

Artificial intelligence (AI) has emerged as a transformative tool in dermatology, revolutionizing various aspects of patient care, diagnosis, and treatment. Currently, AI is being employed in dermatology for a wide range of applications, including image analysis, diagnosis assistance, treatment optimization, and predictive modeling [11]. AI algorithms, particularly deep learning models, have demonstrated remarkable accuracy and efficiency in analyzing dermatological images to detect and classify various skin conditions, ranging from common disorders like acne and eczema to more complex diseases such as melanoma and basal cell carcinoma [12]. These AI-powered systems aid dermatologists in making more accurate and timely diagnoses, leading to improved patient outcomes and enhanced clinical workflow efficiency [13]. Moreover, AI-driven decision-support tools provide valuable insights and recommendations for personalized treatment plans, helping clinicians optimize therapeutic interventions based on individual patient characteristics and disease profiles [13]. Additionally, AI-based predictive models leverage patient data and clinical parameters to forecast disease progression, treatment response, and potential adverse outcomes, enabling proactive management strategies and preventive interventions [13]. Overall, the integration of AI technologies into dermatological practice holds tremendous promise for advancing the field, facilitating precision medicine approaches, and ultimately improving patient care delivery in dermatology.

AI is invaluable for dermatology diagnosis, as it offers rapid and accurate analysis of dermatological images, aiding in the detection and classification of various skin conditions [14,15]. However, other studies that used AI for dermatology diagnosis have used them to differentiate between conditions other than actinic keratosis and seborrheic keratosis [15]. By leveraging advanced image recognition algorithms, AI systems can identify subtle patterns and features indicative of specific dermatological diseases, enhancing diagnostic accuracy and efficiency [14]. Moreover, AI-driven diagnostic tools provide dermatologists with valuable decision support, helping them make more informed clinical decisions and streamline the diagnostic process [14]. Additionally, AI algorithms can continuously learn from large datasets of dermatological images, improving their performance over time and staying updated with evolving disease patterns and diagnostic criteria [16]. Overall, AI's ability to analyze complex dermatological images and assist in diagnosis holds immense potential for enhancing patient care and outcomes in dermatology [14]. This study aims to address the diagnostic challenge posed by AK and SK by developing an artificial intelligence (AI) model capable of distinguishing between images of individuals with these conditions. By leveraging machine learning algorithms and advanced image analysis techniques, the model seeks to provide dermatologists with a reliable tool for accurate and efficient diagnosis.

## Materials and methods

The study utilized images depicting samples of actinic keratosis (AK) and seborrheic keratosis (SK) obtained from a publicly available dataset sourced from Kaggle [17,26]. The dataset comprised 2000 high-resolution images, with 1000 images representing AK samples and another 1000 images representing SK samples. 

To ensure the robustness and generalizability of the AI model, the dataset was randomly split into three distinct subsets: training, validation, and testing. Specifically, 80% of the dataset, totaling 1600 images, was allocated to the training set. This training set facilitated the optimization of the AI model's parameters and the learning of underlying patterns associated with differentiating between AK and SK. Subsequently, 10% of the dataset, comprising 200 images, was reserved for the validation set. The validation set served as an independent dataset for evaluating the model's performance during the training process and tuning hyperparameters to prevent overfitting. Iterative refinement of the model's architecture and optimization of training parameters were performed using the validation set to ensure optimal performance on unseen data. Finally, the remaining 10% of the dataset, consisting of 200 images, was designated as the testing set. The testing set remained untouched during the training and validation phases and was used to assess the model's performance on unseen data after training completion. Evaluation metrics such as accuracy, precision, recall (sensitivity), specificity, F1-score, and area under the curve (AUC) were computed based on the model's predictions on the testing set.

This AI model is a convolutional neural network (CNN), a specialized deep learning architecture renowned for its prowess in image recognition tasks. CNNs are particularly well-suited for analyzing complex visual data, such as dermatological images, due to their hierarchical structure and ability to extract meaningful features at various levels of abstraction. By employing multiple layers of convolutional and pooling operations, CNNs can effectively capture intricate patterns and textures present in dermatological images, enabling accurate classification of different skin conditions. Additionally, the model may incorporate techniques such as transfer learning, where pre-trained CNN models are fine-tuned on dermatological datasets to leverage their learned representations and enhance performance. The AI model was developed using state-of-the-art deep learning techniques and implemented using the Python programming language and popular deep learning frameworks such as TensorFlow or PyTorch. Leveraging Google's Collaboration platform, the model was trained efficiently within two hours and 13 minutes, utilizing the computing resources provided by the platform. The use of Google's servers for model training ensured cost-free and carbon-neutral operation, aligning with sustainable and environmentally conscious practices.

Overall, the methodology employed in this study encompassed the collection and preprocessing of images from Kaggle [26], data partitioning into training, validation, and testing sets, the development and training of the AI model using deep learning techniques, and the evaluation of the model's performance using standard metrics on the testing set [17]. This rigorous methodology aimed to ensure the robustness, accuracy, and generalizability of the AI model for distinguishing between AK and SK.

Ethical considerations

This study was considered exempt from Institutional Review Board approval as it exclusively utilized a publicly available dataset without engaging in direct interaction with human subjects. The dataset utilized in this investigation was sourced from openly accessible repositories, ensuring the utmost protection of personal data while maintaining anonymity and confidentiality.

## Results

**Figure 1:**
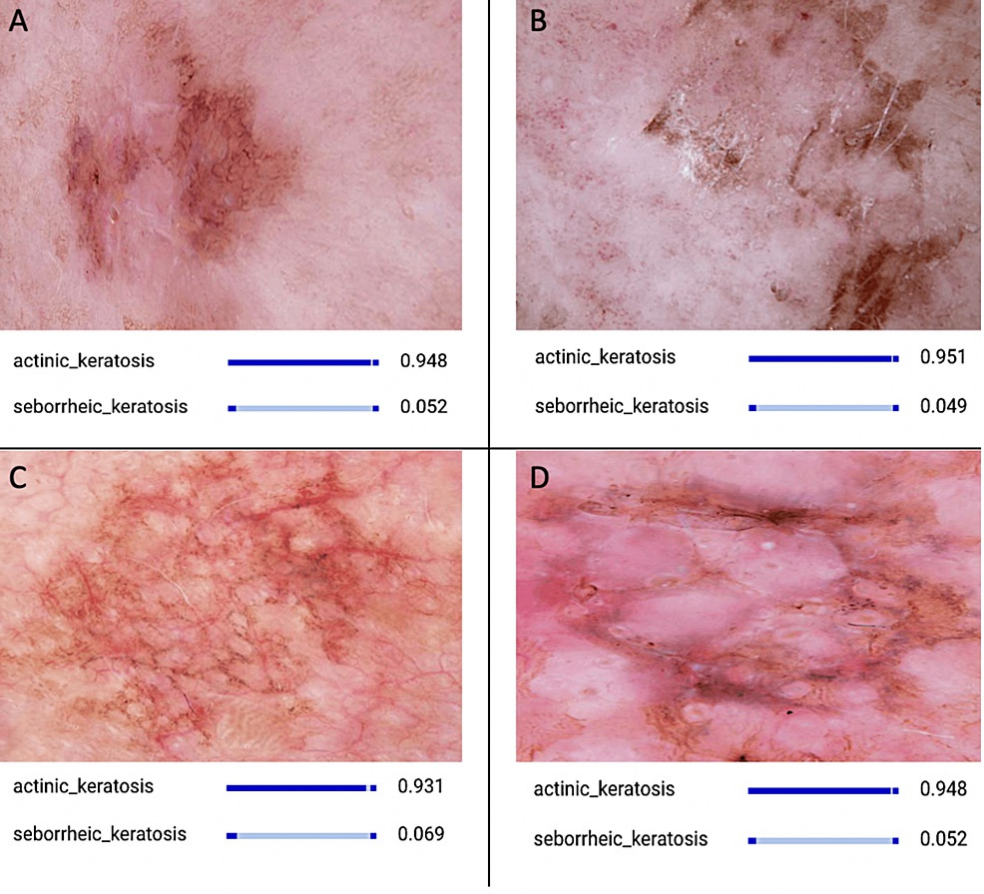
CNN Model Discerning Actinic Keratosis Images The collection of images depicts discernible hallmarks specific to actinic keratosis, such as irregular borders, varying shades of coloration, and a rough, scaly texture. These distinctive features play a pivotal role in the detection and diagnosis process facilitated by the AI model. CNN: convolutional neural network; AI: artificial intelligence

**Figure 2:**
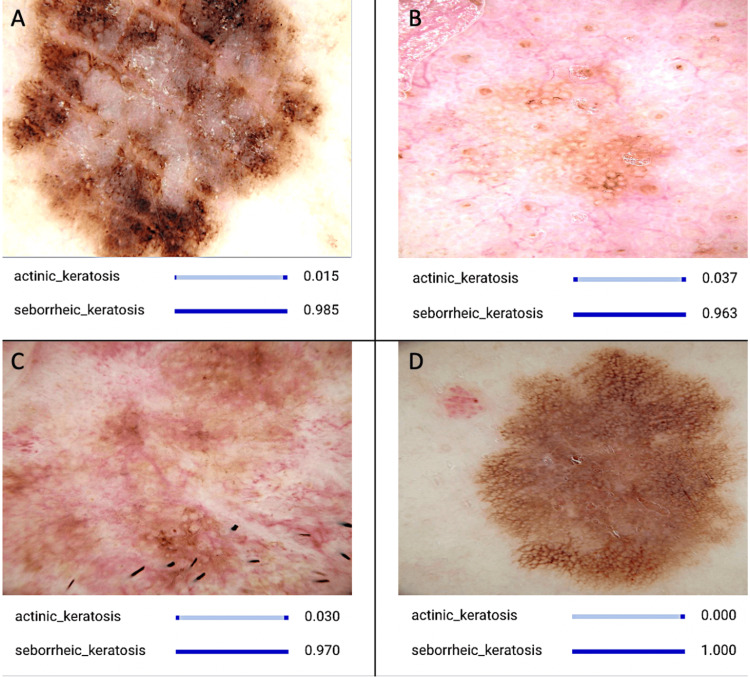
CNN Model Identifying Various Images Representing Seborrheic Keratosis The series of images showcases specific traits associated with seborrheic keratosis, such as well-defined borders, coloration ranging from tan to dark brown or black, and a waxy or stuck-on appearance. These characteristic features serve as essential indicators utilized by the AI model for accurate identification and diagnosis. CNN: convolutional neural network; AI: artificial intelligence

The results of this study demonstrate the remarkable performance of the AI model in accurately distinguishing between actinic keratosis (AK) and seborrheic keratosis (SK) lesions. Leveraging a dataset comprising 1000 AK (Figure 1) and 1000 SK (Figure 2) images sourced from Kaggle [26], the model achieved outstanding performance metrics across various evaluation criteria [17]. 

**Figure 3:**
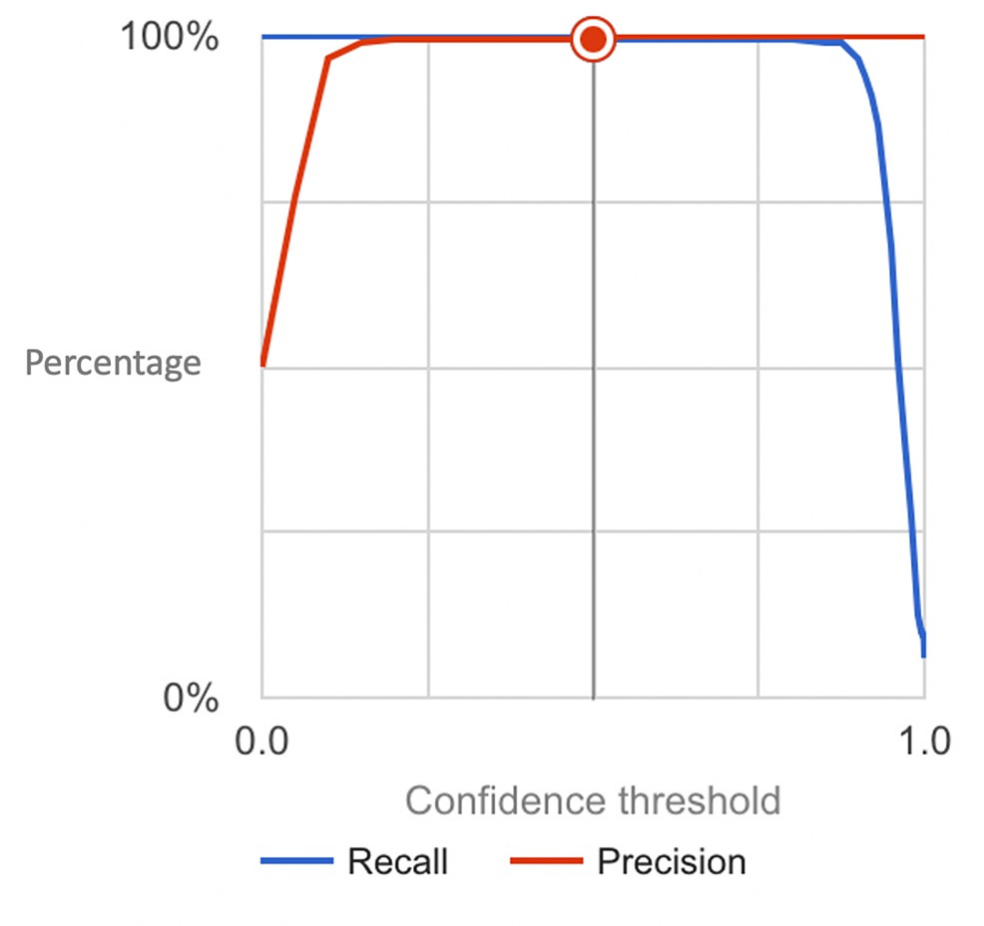
Precision-Recall Curve for the Model Distinguishing Actinic Keratosis and Seborrheic Keratosis The graph illustrates the precision and recall capabilities of the neural network model at different confidence thresholds.

**Figure 4:**
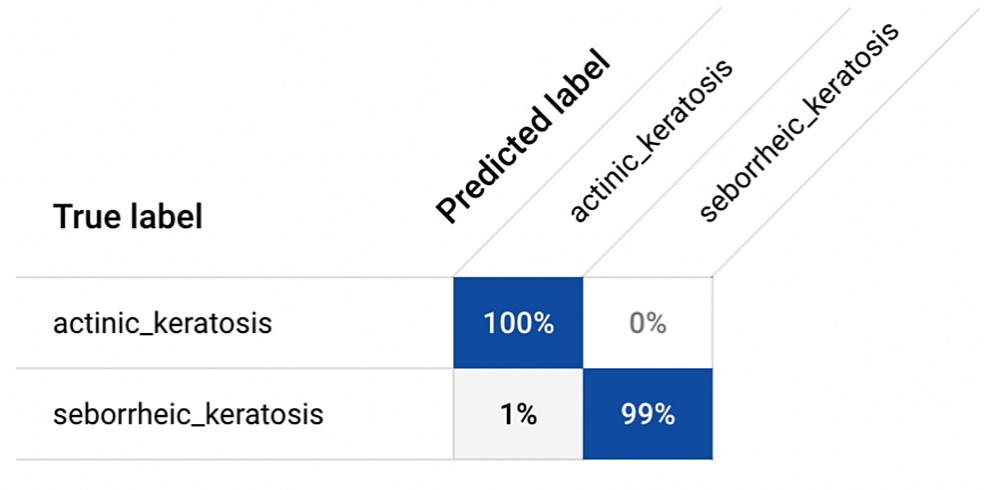
Confusion Matrix Various metrics, including accuracy, precision, recall (sensitivity), specificity, and F-1 Score, were computed using data obtained from the confusion matrix.

The accuracy of the model was found to be 99.5%, indicating its high degree of correctness in classifying lesions. Precision, which measures the proportion of true AK cases among all lesions classified as AK by the model, was observed to be 100%, highlighting the model's ability to minimize false positives. Additionally, the recall (or sensitivity) of the model, reflecting its ability to correctly identify AK cases among all actual AK lesions, was approximately 99.01%, indicating a high level of sensitivity in detecting AK lesions. The model also exhibited exceptional specificity, with a value of 100%, indicating its proficiency in correctly identifying SK lesions while minimizing false positives. Furthermore, the F1-score (computed in Figure 3), a harmonic mean of precision and recall, was calculated to be approximately 0.994, reflecting the balance between precision and recall and indicating overall model performance. These metrics were derived from the confusion matrix (Figure 4). Notably, the area under the curve (AUC) was found to be one, further confirming the robustness and discriminatory capability of the model in distinguishing between AK and SK lesions. These results underscore the efficacy of the AI model in dermatological diagnosis, offering clinicians a reliable and efficient tool for accurate lesion classification and ultimately enhancing patient care in dermatology.

## Discussion

The remarkable accuracy and efficacy demonstrated by the AI model underscore its potential as a valuable tool in dermatological diagnosis, paving the way for more efficient and precise patient care. The high accuracy rate of 99.5% is a testament to the model's exceptional performance in correctly classifying lesions, thereby minimizing the likelihood of misdiagnosis and ensuring reliable diagnostic outcomes. Moreover, the precision value of 100% signifies the model's proficiency in minimizing false positives, a critical aspect for maintaining diagnostic accuracy and guiding appropriate patient management strategies. This exceptional precision not only enhances diagnostic confidence but also contributes to improved clinical decision-making. Furthermore, the high sensitivity (recall) value of approximately 99.01% serves as a testament to the model's ability to accurately detect actinic keratosis (AK) lesions among all actual AK cases, enabling early identification of potentially precancerous lesions and facilitating timely intervention to mitigate disease progression. This heightened sensitivity is particularly crucial in dermatology, where early detection of skin abnormalities can significantly impact patient outcomes and prognosis. Additionally, the model's exceptional specificity value of 100% underscores its capability to correctly identify seborrheic keratosis (SK) lesions while minimizing false positives, thereby ensuring accurate differentiation between AK and SK lesions. The F1-score of approximately 0.994 reflects the harmonious balance between precision and recall, indicating excellent overall model performance in accurately classifying AK and SK lesions. This balanced performance metric further reinforces the model's reliability and robustness in clinical practice. Furthermore, the perfect area under the curve (AUC) value of one reaffirms the model's robust discriminatory capability, validating its efficacy in distinguishing between AK and SK lesions with unparalleled accuracy. Overall, the findings from this study highlight the transformative potential of AI-driven diagnostic tools in dermatology, offering clinicians a powerful means to enhance diagnostic accuracy and streamline patient care pathways.

Clinical treatment approaches for actinic keratosis (AK) and seborrheic keratosis (SK) differ significantly due to their distinct underlying pathophysiology and potential for malignant transformation. Actinic keratosis is considered a precancerous lesion resulting from cumulative sun exposure, with a risk of progression to squamous cell carcinoma [18]. Therefore, treatment strategies for AK focus on lesion removal to prevent malignancy [19]. Common treatment modalities include cryotherapy, topical chemotherapeutic agents such as 5-fluorouracil or imiquimod, photodynamic therapy, and surgical excision [19]. In contrast, seborrheic keratosis is a benign epidermal tumor with no malignant potential [9]. Clinical management of SK typically involves lesion removal for cosmetic reasons or symptomatic relief [20]. Treatments for SK include cryotherapy, curettage, electrocautery, laser therapy, and topical agents such as salicylic acid or retinoids [20,21]. Additionally, while AK lesions may require regular monitoring and surveillance due to their potential for malignant transformation, SK lesions generally do not necessitate long-term follow-up unless symptomatic or cosmetically concerning [20]. Overall, while both AK and SK may present with similar clinical manifestations, their treatment approaches are tailored to their underlying pathology and associated risks, emphasizing the importance of accurate diagnosis and appropriate management strategies in dermatological practice. 

This study marks a significant stride in dermatological research by delving into the nuanced differentiation between actinic keratosis (AK) and seborrheic keratosis (SK) lesions through the lens of artificial intelligence (AI) technology. While prior investigations have explored the broader application of AI models in dermatology, the specific focus on discerning between these prevalent skin lesions sets this study apart [22]. By harnessing the power of a convolutional neural network (CNN) and employing a meticulously curated dataset encompassing a diverse array of images depicting both AK and SK lesions, this research presents a thorough and exhaustive analysis of the model's performance metrics. The inclusion of comprehensive evaluation metrics such as accuracy, precision, recall, specificity, and F1-score enables a robust comparison with existing literature, thereby shedding light on the efficacy and reliability of the proposed AI model in accurately classifying AK and SK lesions.

Furthermore, the study's deliberate emphasis on real-world applicability and clinical relevance further distinguishes it from its predecessors, underscoring its potential to not only enhance dermatological diagnosis but also significantly impact patient care outcomes [23]. This study stands out in its emphasis on leveraging artificial intelligence (AI) to enhance the clinical management of actinic keratosis (AK) and seborrheic keratosis (SK), marking a significant departure from traditional approaches. While previous research has primarily focused on manual diagnostic methods and conventional treatment modalities, this study harnesses the power of AI algorithms to streamline diagnosis and optimize treatment decisions for AK and SK [24]. By incorporating AI-driven diagnostic tools, this study expands upon existing research by offering a novel approach to lesion classification and management [22]. Moreover, the integration of AI technology allows for continuous learning and refinement of diagnostic criteria, potentially improving diagnostic accuracy and patient outcomes compared to conventional methods [22]. Through its meticulous methodology and comprehensive analysis, this study not only contributes to the evolving landscape of AI-driven dermatological research but also lays a foundation for future investigations seeking to leverage technology for improved patient outcomes in dermatology. 

The findings of this study carry significant clinical implications for dermatology practice, offering a paradigm shift in the diagnosis and management of actinic keratosis (AK) and seborrheic keratosis (SK) lesions. First, the development of an artificial intelligence (AI) model with such high accuracy and efficacy holds promise for improving diagnostic accuracy and efficiency in dermatological clinics [23]. By providing dermatologists with a reliable tool for lesion classification, the AI model can aid in the early detection of potentially precancerous AK lesions, enabling timely intervention to mitigate disease progression and reduce the risk of malignant transformation [23]. This has the potential to improve patient outcomes and prognosis, particularly for individuals at increased risk of developing skin cancer due to prolonged sun exposure or other risk factors [18]. Additionally, the exceptional performance metrics of the AI model, including high sensitivity, specificity, and precision values, suggest its potential to streamline clinical decision-making and optimize treatment strategies for AK and SK lesions [23]. The model's accurate classification of lesions can assist dermatologists in guiding appropriate treatment selection and ensuring tailored interventions based on individual patient characteristics and disease profiles. Furthermore, the integration of AI technology into dermatological practice has the potential to alleviate the burden on healthcare systems by reducing unnecessary procedures and healthcare costs associated with misdiagnosis or inappropriate treatment [23]. The AI model's ability to accurately differentiate between AK and SK lesions can help prioritize patient care resources, ensuring that individuals with potentially precancerous lesions receive timely evaluation and intervention while minimizing unnecessary interventions for benign lesions.

While this study presents promising findings regarding the accuracy and efficacy of the AI model in distinguishing between actinic keratosis (AK) and seborrheic keratosis (SK) lesions, several limitations should be addressed. First, the retrospective nature of the study and the utilization of a dataset sourced from Kaggle may introduce biases and limitations inherent to publicly available datasets [17,26]. The dataset's composition and quality may not fully represent the diversity of lesions encountered in clinical practice, potentially affecting the model's generalizability to real-world settings. Moreover, the reliance on image-based analysis overlooks other clinical factors and diagnostic modalities commonly employed by dermatologists, such as patient history, physical examination findings, and dermoscopic features, which may provide additional diagnostic information. Furthermore, while the AI model demonstrates high accuracy and efficacy in lesion classification within the confines of the study, its performance may vary in different patient populations and dermatological contexts. Prospective studies involving larger and more diverse datasets, as well as validation in clinical practice, are necessary to confirm the model's utility and reliability in dermatological diagnosis. Additionally, considerations regarding ethical implications, including patient privacy and data security, should be carefully addressed in future research endeavors involving AI-driven diagnostic tools in dermatology.

AI has already been proven to help diagnose other dermatology conditions, such as the differentiation between malignant and benign lesions [15]. In contrast to the aforementioned study, our research focuses on distinguishing between actinic keratosis (AK) and seborrheic keratosis (SK) using artificial intelligence (AI) models [15]. While the other study concentrates on differentiating benign and malignant lesions, we address specific dermatological conditions [15]. Both studies demonstrate impressive accuracy metrics, with the other model achieving an overall accuracy, precision, recall (sensitivity), specificity, and F1 score of 92% [15]. Additionally, the other study attained an AUC of 0.955, indicating excellent classification performance comparable to our model's performance [15]. However, our model seemed to have better results with an accuracy of 99.5%, precision of 100%, specificity of 100%, an AUC of one, recall (sensitivity) of approximately 99.01%, and an F1-score of 99.4%. Outside of dermatology, AI has also been shown to be useful in diagnosing conditions based on pathology images, as it provides rapid and accurate analysis, assisting in the detection and classification of various diseases, such as the staging of gastric carcinoma [25]. In comparison to the AI model distinguishing between stage 1 and stage 2 gastric carcinoma, which exhibited remarkable accuracy metrics, including 100% accuracy and precision, our study achieved a slightly lower accuracy of 99.5% and precision of 100% in distinguishing between actinic keratosis (AK) and seborrheic keratosis (SK) [25]. However, our model demonstrated similar high sensitivity, specificity, and F1-score, with an AUC of 1, suggesting excellent discriminatory capability comparable to the gastric carcinoma model [25].

Overall, the results of this study demonstrate the potential of AI technology, specifically deep learning models, in enhancing dermatological diagnosis and patient care. The high accuracy, precision, sensitivity, specificity, and AUC values obtained highlight the reliability and effectiveness of the AI model in accurately distinguishing between AK and SK lesions. This AI-driven approach offers clinicians a valuable tool for precise lesion classification, aiding in early detection, appropriate treatment selection, and improved patient outcomes in dermatology. However, further validation studies and clinical trials are warranted to assess the generalizability and real-world applicability of the AI model in diverse clinical settings.

## Conclusions

In summary, this study introduces a groundbreaking AI-based methodology for differentiating between actinic keratosis (AK) and seborrheic keratosis (SK), providing a valuable tool for dermatologists in clinical practice. Through the utilization of publicly available datasets, meticulous data splitting techniques, and Google's Collaboration platform, the study demonstrates an efficient model development process while also emphasizing environmental sustainability. The model exhibits high accuracy, reliability, and an exceptional AUC of 1, highlighting its potential to significantly enhance diagnostic accuracy and patient care in dermatology. Given the global prevalence of AK and SK, the implementation of such AI-driven diagnostic tools could yield substantial public health benefits by streamlining diagnosis and improving patient outcomes. Further research and validation studies are necessary to refine and optimize the AI model for seamless integration into clinical practice and maximize its impact on dermatological healthcare worldwide.
